# Experience Matters: The Effects of Hypothetical versus Experiential Delays and Magnitudes on Impulsive Choice in Delay Discounting Tasks

**DOI:** 10.3390/brainsci9120379

**Published:** 2019-12-16

**Authors:** Catherine C. Steele, MacKenzie Gwinner, Travis Smith, Michael E. Young, Kimberly Kirkpatrick

**Affiliations:** 1 Department of Food, Nutrition, Dietetics, and Health, Kansas State University, Manhattan, KS 66506, USA; 2 Department of Psychological Sciences, Kansas State University, Manhattan, KS 66506, USAkirkpatr@ksu.edu (K.K.)

**Keywords:** impulsive choice, delay discounting, experiential discounting task (EDT)

## Abstract

Impulsive choice in humans is typically measured using hypothetical delays and rewards. In two experiments, we determined how experiencing the delay and/or the reward affected impulsive choice behavior. Participants chose between two amounts of real or hypothetical candy (M&Ms) after a real or hypothetical delay (5–30 s), where choosing the shorter delay was the impulsive choice. Experiment 1 compared choice behavior on a real-delay, real-reward (RD/RR) task where participants received M&Ms after experiencing the delays versus a real-delay, hypothetical-reward (RD/HR) task where participants accumulated hypothetical M&Ms after experiencing the delays. Experiment 2 compared the RD/HR task and a hypothetical-delay, hypothetical-reward (HD/HR) task where participants accumulated hypothetical M&Ms after hypothetical delays. The results indicated that choices did not differ between real and hypothetical M&Ms (Experiment 1), and participants were less sensitive to delay and more larger-later (LL)-preferring with hypothetical delays compared to real delays (Experiment 2). Experiencing delays to reward may be important for modeling real-world impulsive choices where delays are typically experienced. These novel experiential impulsive choice tasks may improve translational methods for comparison with animal models and may be improved procedures for predicting real-life choice behavior in humans.

## 1. Introduction

Impulsive choice is a widely studied construct that measures an organism’s propensity to opt for a smaller-sooner (SS, impulsive) outcome rather than wait for a larger-later (LL, self-controlled) outcome. Preference for the SS option is suboptimal under conditions where waiting for the LL option provides greater long-term outcomes [1]. Suboptimal preference for the SS option in humans is associated with other maladaptive behaviors such as substance use disorders [2], pathological gambling [3], obesity [4], and Attention Deficit Hyperactivity Disorder (ADHD) [5,6] and is considered a trans-disease process [7,8,9]. The predictive power of impulsive choice for maladaptive behaviors makes it a prime target to understand the processes that may produce such behaviors and the development of interventions to treat those processes.

Impulsive choice is a robust construct that can be assessed through a variety of procedures, including experiential and hypothetical tasks [9,10]. Performance in the experiential and hypothetical tasks have both successfully predicted maladaptive behaviors and real-life outcomes between individuals. A well-known experiential impulsive choice task is the delayed gratification task (i.e., the “marshmallow test” [11]) where children could earn two rewards if they waited for a period of time and resisted consuming the single reward tempting them at the moment. The ability to delay gratification in children at 4–5 years of age predicted their SAT scores and social skills several years later and even predicted weight status 30 years later [9]. Alternatively, hypothetical tasks are a common way to measure impulsive choice by presenting participants with the choice between a larger amount of money after a delay or a smaller amount of money available now [10,12]. The participants do not receive the reward and they do not experience the delay, but they are asked to make choices as if they were real. While this approach lacks the experiential element that the delayed gratification (i.e., marshmallow) tasks offers, it allows researchers to collect data on numerous choices to obtain a more graded measure of impulsive choice in comparison to the one-shot choice of the delayed gratification test. The impulsive choice task concurrently offering two options is sometimes referred to as a delay discounting task because the preference for the delayed rewards is observed to decrease (i.e., be discounted) as the delay to their receipt increases [13]. The accumulated evidence consistently links high rates of delay discounting for hypothetical rewards with a myriad of disorders and maladaptive behaviors (similar to delay of gratification), and high discounting (i.e., impulsive choice) is considered a trans-disease process [7,8,14].

Delay discounting tasks are often hypothetical, but some studies have attempted to validate whether choice for hypothetical rewards approximate choices for real-life rewards. To ensure that the participants’ hypothetical choices reflect what they would choose if they were going to experience the actual delay and reward, researchers have used an impulsive choice task with potentially real rewards (e.g., [15]). The goal is to encourage participants to respond as though they are receiving each of their chosen rewards by providing the real outcome from one of their choices. It has been established that in many cases hypothetical and potentially real monetary rewards can result in similar choice functions [12,16,17]. However, there is limited and mixed evidence as to whether consumables (e.g., food) are equivalently discounted as real and hypothetical rewards. One study found that potentially real cigarettes were discounted more steeply than hypothetical cigarettes [18], but another study found that hypothetical and potentially real food rewards were not discounted differently [16]. In addition, task type may interact with other variables. For example, while body fat percentage and impulsive choice are related [19], this relationship is clearer when the impulsive choice task includes food choices or real money choices as opposed to hypothetical monetary choices [19,20,21]. In addition, individuals with ADHD only showed improvements in self-controlled choices with methylphenidate when the outcomes were experienced [22]. The mixed success of hypothetical impulsive choice tasks in detecting the effects of interest demonstrate their limitations as methods to study impulsivity.

One challenge with the hypothetical food and money tasks is that individuals may fail to accurately imagine how the consequences of their current choices may alter future choices. Thus, hypothetical choices may not be as readily modified by experience because they do not involve any feedback. For example, if you gave a participant a choice between one hypothetical bite of food immediately or several hypothetical bites after 60 s, the participant may frequently choose the larger-later option without considering how distressingly long 60 s can seem. The actual experience of an aversive 60-s delay may change future choices. Individuals may also fail to consider how several bites may accumulate over trials and satiety may eventually set in.

Experiential discounting tasks (EDTs) for use with human participants have been developed to address some of the limitations of the hypothetical tasks. In EDTs, participants receive the rewards and experience the delays, such as having to wait 30 s before they can bank a monetary reward. These tasks are, thus, more similar to nonhuman animal tasks [23]. Although they may represent an improvement over hypothetical tasks, EDTs have received criticism for several major deviations from the nonhuman animal procedures [24]. Specifically, previously developed EDTs (1) have probabilistic outcomes, whereas nonhuman animal procedures have certain outcomes; (2) do not impose an inter-trial interval after the choice; and (3) result in points as opposed to rewards such as food that can be consumed during the session. Smits et al. [24] reported that their EDT had poor cross-test reliability with a typical hypothetical discounting assessment. They also reported that performance was highly correlated with boredom proneness. Thus, performance on the EDT, offering small monetary rewards per trial, might be measuring task boredom rather than an impulsive disposition.

Given these important considerations for measuring impulsive choice behavior coupled with the criticisms of current experiential discounting tasks, the goal of the present experiments was to create a real-delay, real-reward impulsive choice task for food that addressed the criticisms outlined in Smits et al. [24] by delivering certain outcomes and including an inter-trial interval. Unlike the Smits et al. [24] study, the real reward was a consumable reward in the form of mini M&Ms. The current experiments examined how experiencing the delay and/or reward affected choice behavior. Experiment 1 compared a real-delay, real-reward (RD/RR) task to a real-delay, hypothetical-reward (RD/HR) task. The hypothetical reward was similar to the previous EDTs in that the reward was not actually experienced. Thus, the design of Experiment 1 was targeted to address the previous criticisms of experiential tasks. Experiment 2 compared a real-delay, hypothetical-reward (RD/HR) task to a hypothetical-delay, hypothetical-reward (HD/HR) task. This experiment was designed as a companion to Experiment 1 by testing the importance of the experience of delays. We tested this effect with hypothetical rewards to assess this issue in the context of the conditions of previous experiential tasks, which also used hypothetical rewards. Experiment 2 also assessed the association of real and hypothetical delay impulsive choice with boredom proneness and sensation seeking to determine whether these other variables may explain task performance. A hypothetical, monetary impulsive choice questionnaire [25] was used as a benchmark for comparison to a standard hypothetical choice task in both experiments. This set of experiments provided a novel way to understand how experiencing delay and reward impacted impulsive choices, which can help uncover the critical variables that affect real world, impulsive choices.

## 2. Materials and Methods

### 2.1. Subjects

Participants were recruited in Riley County, KS, USA, through general psychology courses, fliers, and word of mouth. The participants’ characteristics across the experiments are described in Table 1. Because of their novel design, we did not have a good basis for conducting a predictive power analysis. Instead, our sample size was informed by published studies using similar repeated measures designs [24]. The study description indicated that they would make choices for M&Ms. Liking of chocolate was an inclusion criterion. Participants were compensated with US$5 or course credit. Individuals were excluded from participation if they were under the age of 18, nonfluent in English, diagnosed with a metabolic disorder (e.g., diabetes) or eating disorder, had extremely restrictive eating habits (e.g., no carb diet), were taking medication that could impact hunger/appetite, or were a smoker. Participants self-reported that these exclusion criteria did not apply. The studies were approved by the Institutional Review Board at Kansas State University.

### 2.2. Procedures

**Figure 1:**
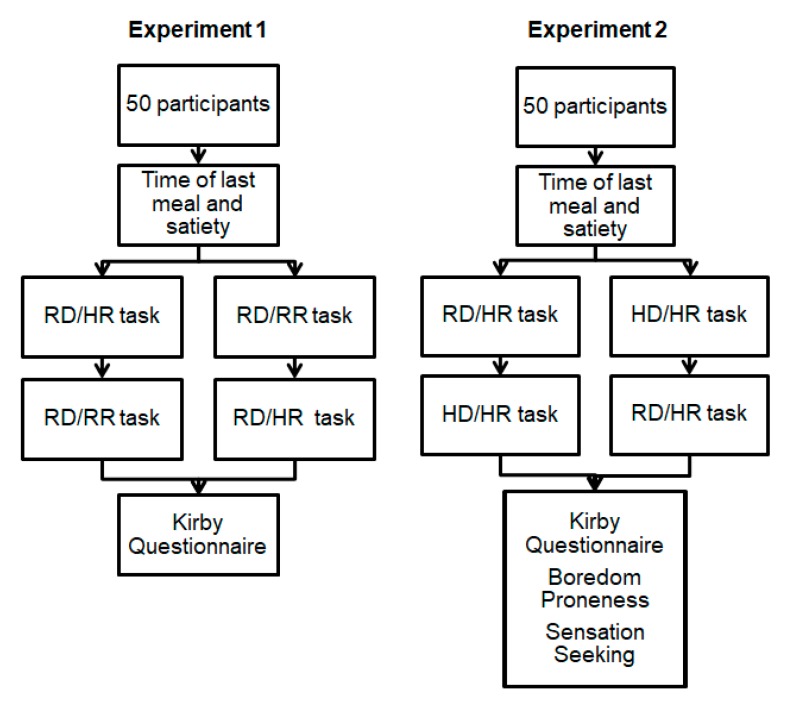
Flowchart of the progression through the session in Experiments 1 and 2. Participants were randomly assigned to different orders of task presentation at points where the chart branches off. RD/RR = real-delay, real-reward. RD/HR = real-delay, hypothetical-reward. HD/HR = hypothetical-delay, hypothetical-reward.

An overview of the procedure in the two experiments is shown in Figure 1. For both experiments, participants were asked to not eat or drink for at least 4 h before the testing session. Upon arrival to the laboratory, participants read the informed consent form before indicating the time since their last meal and their subjective hunger on the Holt Satiety Index. Participants were also asked to “circle one of the lines for how you are feeling right now” on the Holt Satiety Index, which ranges from extremely hungry to extremely satisfied [26]. Participants were not asked to report how hungry they were to avoid biasing them toward the hungry side of the scale.

Participants then completed two impulsive choice tasks. In Experiment 1, participants were randomly assigned to complete the real-delay, real-reward (RD/RR) or the real-delay, hypothetical-reward (RD/HR) impulsive choice task first before crossing over to complete the other task. Next, the participants completed a demographic questionnaire and the Kirby questionnaire. In Experiment 2, participants were randomly assigned to complete the RD/HR or the hypothetical-delay, hypothetical-reward (HD/HR) impulsive choice task before crossing over to complete the other task. Next, the participants completed a demographic questionnaire, the Kirby questionnaire, the Boredom Proneness Scale, and the Sensation Seeking Scale.

### 2.3. Novel Impulsive Choice Tasks

#### 2.3.1. Task Overview

**Figure 2:**
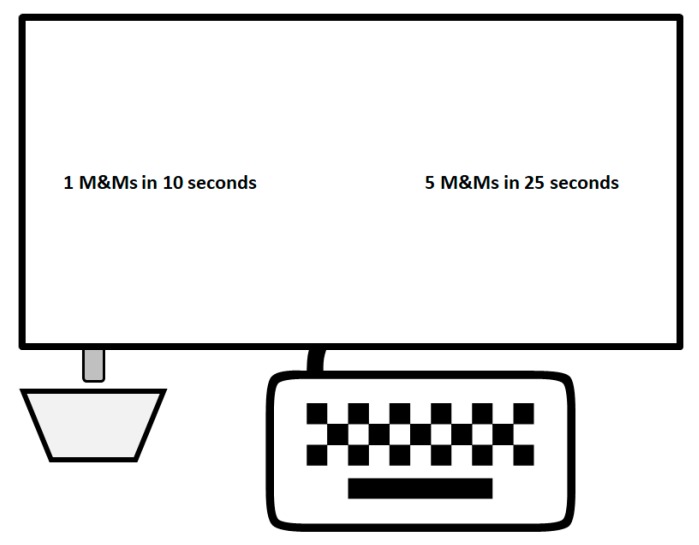
Illustration of the computer setup. Computer monitor screen displayed the two choices, a keyboard was used to make a choice (F keypress for left selection, J keypress for right selection), and a dish collected mini M&Ms delivered via a tube from a dispenser hidden behind the monitor.

To measure impulsive choice behavior, participants chose between SS and LL rewards, with the SS choice characterized as the impulsive choice. Participants sat in front of a computer and they were presented with 25 choices, where the SS option delivered 1–4 mini M&Ms after a short delay and the LL option delivered 4 or 5 mini M&Ms after a longer delay (Figure 2). The delays ranged from 5 to 30 s. The number of mini M&Ms and the delay to reward were indicated on the screen (e.g., 1 M&M in 5 s or 5 M&Ms in 25 s). Table A1 in Appendix A depicts the combinations of delay and magnitude ratios (delay ratio = SS delay/LL delay; magnitude ratio = SS magnitude/LL magnitude). Each participant experienced each magnitude/delay ratio combination once. The choice parameters were distributed equally across two blocks, as consumption during the real food choice task may affect choices in the second block of the task [27]. Each block included at least one trial with each delay and magnitude ratio. The three impulsive choice tasks differed in whether participants waited the delay and/or received the actual reward.

During the real-delay, hypothetical-reward (RD/HR) impulsive choice task, the participants chose their preferred option and waited the delay before they were able to “store” the pieces of candy on the screen. The participants did not receive any food rewards to consume, but they could see the number of mini M&Ms they had earned in their store. They were asked to make the choice as though they were going to receive those M&Ms after the specified amount of time. During the real-delay, real-reward (RD/RR) impulsive choice task, the participants obtained the actual food reward after they experienced the delay. The participants received their choice and consumed the food reward, but they could not see the total number of mini M&Ms they had earned. Participants could earn a maximum of 120 mini M&Ms (~1 oz; 141 kcal). The mini M&Ms were delivered through a feeder (see apparatus) following the first keypress after the delay elapsed. Participants were not required to consume the M&Ms before proceeding to a new trial, but they were not allowed to accumulate the M&Ms to consume after the session. During the hypothetical-delay, hypothetical-reward (HD/HR) impulsive choice task, the participants chose their preferred option, but they did not have to wait the delay before they were able to “store” the pieces of candy on the screen.

#### 2.3.2. Instructions

Upon arrival, participants were asked to set aside their phone and watches, and there were no clocks in the room to encourage reliance on their internal timing mechanisms when making and waiting for their choices. The instructions were as follows for the real-delay impulsive choice tasks: “During this 45-min session, you are going to make a bunch of choices. You will be presented with two options on the screen and you will choose which ever option that you want. After making that choice by pressing “F” or “J”, you must wait that amount of time and press that key again to receive the reward. You may press that key as many times as you want, but the response after that amount of time will result in the food delivered to the bowl (or store).” Participants were not told when the delay had elapsed, rather, they had to estimate the amount of time that had elapsed. Participants were required to make a response to deliver the reward to ensure that they were actively anticipating and timing the reward deliveries. A 30-s intertrial interval (ITI) commenced following delivery of the reward during which a blank white screen was shown. The ITI and fixed number of trials ensured that choosing the LL was the optimal choice assuming that maximum reward earning was the participant’s goal. Details about the length of the task were ambiguous. Participants did not know if the session was a set amount of time or whether their choice influenced how long they were in the session. If participants knew that there were a fixed number of trials, they might be more likely to choose the SS option to get out of the session sooner. In addition, if participants knew there was a fixed session length, they might be more likely to choose the LL option because they would have to stay there the same amount of time regardless.

For the RD/RR impulsive choice task, participants were additionally instructed that “There will be a blank screen following your choice. This time is intended for you to eat the M&Ms. The next choice will pop up automatically. There is water here for you in case you need it.” For the RD/HR impulsive choice task, participants were additionally instructed that “There will be a blank screen following your choice, and the next choice will pop up automatically.” Water was also available to participants during the RD/HR task in Experiment 1. The HD/HR task did not require that the participants wait the delay or experience the ITI. The participants were simply instructed to press twice (once to choose and once to receive the reward). Therefore, the minimum effort requirement was the same for all tasks.

#### 2.3.3. Apparatus

The choice tasks were conducted on a Dell computer. MATLAB 2018a controlled the experimental procedures and collected data. During the RD/RR choice task, the pieces of candy were delivered through a feeder (Med-Associates, St. Albans, VT, USA, ENV-203-MINI) into a small plastic bowl. The feeder was controlled by a transistor to transistor logic interface coupled with a 28V DC adapter (Med-Associates, St. Albans, VT, USA, SG-230R). The adapter connected the feeder, a DC power supply (Med-Associates, St. Albans, VT, USA, SG-500T), and a digital input/output card (National Instruments, Austin, TX, USA; PCIe-6509). The feeder was controlled using the Data Acquisition Toolbox in MATLAB 2018a with millisecond timing precision.

### 2.4. Questionnaires

#### 2.4.1. Kirby Questionnaire

Participants in both experiments answered a series of 27 questions consisting of choices between immediate (smaller-sooner, SS) and delayed (larger-later, LL) rewards [28]. For example, a choice could be US$15 now versus US$35 in 13 d. A single question appeared on the screen and advanced to the next question after the response occurred; the 27 questions were presented randomly intermixed. Instructions for the choice task were “In the task that follows, you will have the opportunity to choose between different amounts of money available after different delays. The test consists of about 30 questions, such as the following: Would you rather have $10 in 30 days or $2 now? You will not receive any of the rewards that you choose, but you should make your decisions as though you were really going to get the rewards you choose.”

#### 2.4.2. Boredom Proneness Scale (BPS)

Participants in Experiment 2 completed the BPS to determine if the experiential tasks may have measured boredom proneness [29]. The BPS is a 28-item questionnaire, where they read statements and responded on a 7-point Likert scale (1 = strongly disagree, 7 = strongly agree). Statements included “It is easy for me to concentrate on my activities” and “It takes more stimulation to get me going than most people.” Possible scores ranged from 28 to 196, with higher scores indicating greater proneness to boredom (Cronbach’s α = 0.83).

#### 2.4.3. Sensation Seeking Scale (SSS)

Participants in Experiment 2 completed the SSS to determine if the experiential tasks were measuring sensation seeking [30]. The SSS is a 40-item questionnaire where participants read two choices. Participants were asked to choose “the choice which most described your likes or the way you feel.” For example, participants choice between “I like ‘wild’ uninhibited parties” and “I prefer quiet parties with good conversation.” Possible scores ranged from 0 to 40, with higher scores indicating greater sensation seeking (Cronbach’s α = 0.80).

### 2.5. Data Analysis

#### 2.5.1. Impulsive Choice Tasks

For the impulsive choice tasks, repeated measures logistic regressions were conducted to determine differences in the proportion of LL choices made during the impulsive choice tasks. The possible fixed effects included task, order, delay ratio (log-transformed), and magnitude ratio (log-transformed). Delay ratio, magnitude ratio, and task were included in all models. Delay ratio assessed sensitivity to delay, and magnitude ratio assessed sensitivity to magnitude. Impulsive choice should be characterized by greater sensitivity to delay and lower sensitivity to magnitude [31,32]. This analysis technique allows the two aspects of impulsive choice to be characterized. Task was included as a categorical predictor to determine similarities in performance across food choice tasks. A data-driven approach was used to determine if block and task order should be included as a fixed effect. Block was included as categorical predictor to determine if choice behavior changed across the session. Task order was tested as a categorical predictor to determine if it should be included as a fixed effect and was included in models where it improved the model fit. The random effects structure included the delay and magnitude ratios. The random effects were used to obtain individual slope estimates for delay and magnitude sensitivity for the cross-task correlations. Delay and magnitude sensitivity were used to derive a *k* value (delay sensitivity/magnitude sensitivity) for the impulsive choice (logistic *k*) [31,32].

In both experiments, adding block as a predictor did not improve the Akaike Information Criterion (AIC; [33]) so it was not incorporated into the final models. In Experiment 1, the fixed effects included task (RD/HR or RD/RR), delay ratio, and magnitude ratio. Order was not included as a predictor because it did not improve the Akaike Information Criterion (AIC; [33]). In Experiment 2, the fixed effects included task (RD/HR or HD/HR), order (RD/HR first or HD/HR first), delay ratio, and magnitude ratio. Coefficient comparisons using the emmeans package in R were conducted to probe significant interactions. As an exploratory analysis to assess differences in choices at the extreme delay (or magnitude) ratios, the differences in choice behavior at the 0.2 and 0.8 delay (and magnitude) ratios were probed using the emmeans package in R.

#### 2.5.2. Cross-Task Correlations

For the Kirby questionnaire, individual *k* values were determined using the Wileyto et al. [31] logistic regression approach (Kirby *k*). Boredom proneness and sensation seeking were calculated according to the scoring protocols. In Experiment 1, the *k*-values obtained from the Kirby questionnaire were correlated with delay sensitivity, magnitude sensitivity, and *k* derived from the logistic regression. In Experiment 2, delay sensitivity, magnitude sensitivity, logistic regression derived *k*, the *k*-values obtained from the Kirby questionnaire, boredom proneness, and sensation seeking were correlated. All correlations were conducted using the rcorr.adjust function from the RcmdrMisc package in R, where the *p* values were corrected for multiple correlations using the Holm method [34].

## 3. Results

### 3.1. Novel Impulsive Choice Task Comparisons

#### 3.1.1. Experiment 1

**Figure 3:**
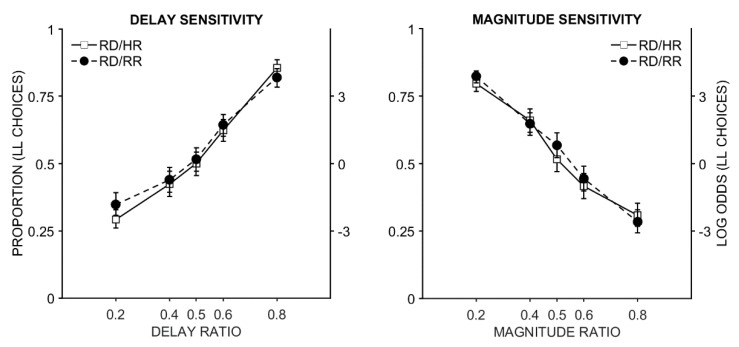
The proportion of LL (self-controlled) choices as a function of the delay ratio (left panel) and magnitude ratio (right panel) on the different tasks. RD/RR = real-delay, real-reward. RD/HR = real-delay, hypothetical-reward. A 0.2 ratio means the LL delay (or magnitude) is 5× longer (or larger) than the SS delay (or magnitude), and a 0.8 ratio means the LL delay (or magnitude) is 1.25× longer (or larger) than the SS delay (or magnitude).

Real reward versus hypothetical reward. The full model outputs are in Table 2. There was a significant main effect of delay and magnitude ratio, such that the proportion of LL choices changed as the delay and magnitude ratios changed. As the delay ratio increased, participants made more LL choices (positive slope) because the delays became more similar. As the magnitude ratio increased, participants made more SS choices because the magnitudes became more similar (negative slope). However, there was no interaction between task (RD/RR and RD/HR) and delay (or magnitude) ratio. This suggests that performance was similar across tasks, consistent with the visual depiction of the data in Figure 3.

#### 3.1.2. Experiment 2

**Figure 4:**
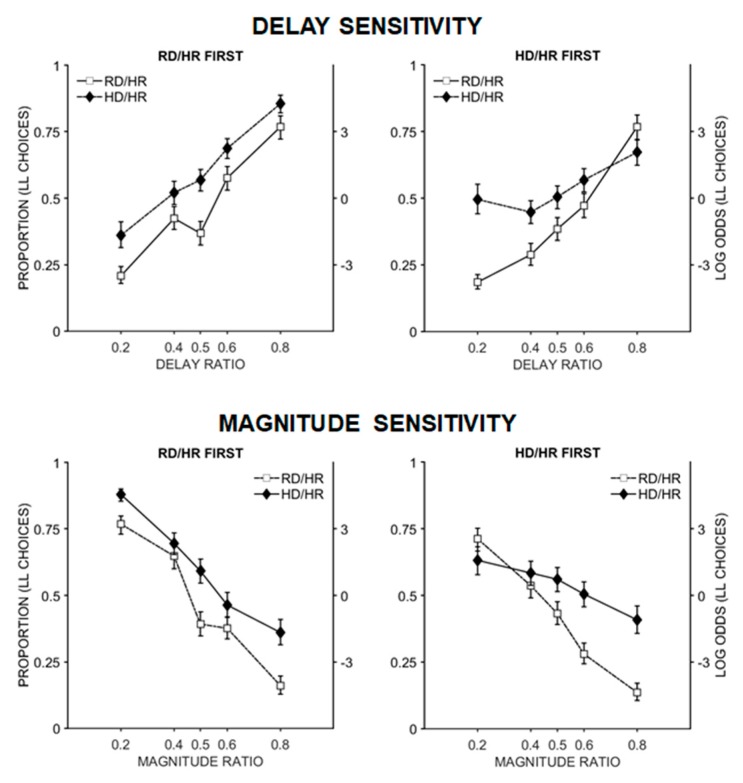
The proportion of LL (self-controlled) choices as a function of the delay ratio (top panels) and magnitude ratio (bottom panels). The top panels show the delay sensitivities on the real delay and hypothetical delay tasks for participants who completed the real delay task first (left) and the hypothetical delay task first (right). The bottom panels show the comparable conditions for magnitude sensitivities. RD/HR = real-delay, hypothetical-reward. HD/HR = hypothetical-delay, hypothetical-reward.

Real delay versus hypothetical delay. The full model output is in Table 3. There was a significant effect of delay and magnitude ratio, such that the proportion of LL choices changed as the delay and magnitude ratios changed. In addition, there was a significant task × order × delay ratio interaction. Coefficient comparisons indicated that for the participants that completed the hypothetical delay task first, delay sensitivity was significantly lower on the hypothetical delay task, *b* = 1.24, than on the real delay task, *b* = 4.28, *z* = 9.30, *p* < 0.001. In addition, delay sensitivity on the real delay task was similar regardless of which task was completed first, *z* = −0.12, *p* = 0.99. Participants who completed the real delay task first were more sensitive to delay on the hypothetical task, *b* = 4.61, than the participants who completed the hypothetical delay task first, *b* = 3.53, *z* = 3.27, *p* = 0.006. This suggests that delay sensitivity is reduced on the hypothetical delay task, especially if participants had not already completed the real delay task (Figure 4). There was a significant task × order × magnitude ratio interaction. Coefficient comparisons indicated that for the participants that completed the hypothetical delay task first, magnitude sensitivity was significantly lower on the hypothetical delay task, *b* = −1.49, than on the real delay task, *b* = −3.61, *z* = −6.91, *p* < 0.001. In addition, magnitude sensitivity on the real delay task was similar regardless of which task was completed first, *z* = −0.78, *p* = 0.86. Participants who completed the real delay task first were more sensitive to magnitude on the hypothetical delay task, *b* = −4.52, than the participants who completed the hypothetical delay task first, *b* = −1.49, *z* = −3.22, *p* = 0.007 (Figure 4). Despite the differences in delay sensitivity and magnitude sensitivity depending on task and order, the *k*-values derived from the sensitivities were similar across tasks regardless of the order that they received the tasks (Table 4). This is likely because delay and magnitude slopes were highly correlated within testing conditions. This demonstrates the importance of separating delay and magnitude sensitivities because dramatically different patterns of behavior, as seen in Figure 4, can result in similar *k*-values.

Coefficient comparisons were conducted to probe the differences at 0.2 and 0.8 delay and magnitude ratios. For the delay ratio functions, participants who completed the real delay task first (Figure 4, top left) made significantly more LL choices during the hypothetical delay task compared to the real delay task when the delay ratio was 0.2, *z* = −5.35, *p* < 0.0001. However, they did not differ when the delay ratio was 0.8, *z* = −1.77, *p* = 0.29. Participants who completed the hypothetical delay task first (Figure 4, top right) made significantly more LL choices during the hypothetical delay task compared to the real delay task when the delay ratio was 0.2, *z* = −9.13, *p* < 0.001, and they made more SS choices during the hypothetical delay task compared to the real delay task when the delay ratio was 0.8, *z* = 5.35, *p* < 0.001.

For the magnitude ratio functions, participants who completed the real delay task first (Figure 4, bottom left) made significantly more LL choices during the hypothetical delay task compared to the real delay task when the magnitude ratio was 0.2, *z* = −3.07, *p* = 0.01, and when the magnitude ratio was 0.8, *z* = −4.37, *p* < 0.001. Participants who completed the hypothetical delay task first (Figure 4, bottom right) made significantly more SS choices during the hypothetical delay task compared to the real delay task when the magnitude ratio was 0.2, *z* = 3.19, *p* = 0.008, and they made more LL choices during the hypothetical delay task compared to the real delay task when the magnitude ratio was 0.8, *z* = −9.40, *p* < 0.001.

### 3.2. Cross-Task Correlations

In Experiment 1, delay and magnitude sensitivity were significantly correlated for the RD/RR and RD/HR impulsive choice tasks. However, the *k*-values obtained from the Kirby questionnaire did not correlate very well with delay sensitivity, magnitude sensitivity, or *k* derived from performance on the real delay impulsive choice task (Table 5). In Experiment 2, delay sensitivity and magnitude sensitivity were highly correlated; however, no other correlations were significant after correcting for multiple correlations (Table 6).

## 4. Discussion

This set of experiments established a novel impulsive choice task for food that is an improvement on the one-shot marshmallow task as it allows for graded choice measurements across multiple parameters. These experiments were designed to improve translational efficacy between rodents and humans and to further the understanding of the effects of experiential outcomes on human impulsive decision-making. Impulsive choice tasks in rodents involve waiting the delays and consuming the rewards, while impulsive choice tasks in humans often utilize hypothetical delays and rewards. Two experiments were conducted to determine the effects of experiencing the delay and/or magnitude of the reward on choice behavior. Repeated measures logistic regressions were used to parse different aspects of choice: sensitivity to delay and sensitivity to magnitude [32,35,36]. Experiment 1 compared impulsive choice on a task where participants experienced the delay and consumed the rewards (real-delay, real-reward) to impulsive choice on a task where participants experienced the delay but did not consume the rewards (real-delay, hypothetical-reward). The results demonstrated that participants displayed nearly identical sensitivity to delay and magnitude of the reward on both tasks. Further, participants responded similarly regardless of whether they received the reward, suggesting that the experience of reward outcomes may not be necessary for people to accurately imagine their preferences. The similarity in performance is key for future research where it might be difficult or impractical for food to be consumed. For example, delivering hypothetical rewards following actual delays could feasibly be used in imaging studies.

Experiment 2 compared impulsive choices on a task where participants experienced the delay but did not consume the rewards (real-delay, hypothetical-reward) to impulsive choices on a task where participants did not experience the delay and did not consume the rewards (hypothetical-delay, hypothetical-reward). The results indicated that impulsive choices depended on the order in which the tasks were experienced. Participants who completed the hypothetical-delay, hypothetical-reward impulsive choice task first were not very sensitive to delay or magnitude. This suggests that they were not able to accurately imagine how the delays would affect their choices. However, if participants experienced the real-delay, hypothetical-reward impulsive choice task first, they were sensitive to delay and magnitude on the hypothetical-delay, hypothetical-reward impulsive choice task. This suggests that participants were able to calibrate their understanding of how delays affected their preferences after having experience with the delays. Interestingly, hypothetical delays reduced magnitude sensitivity in addition to reducing delay sensitivity. This demonstrates that magnitude discrimination judgments in delay discounting tasks may not get processed independently from the delay contingencies. The current experiment did not test a hypothetical-delay, real-reward impulsive choice task, and it is possible that experiencing the rewards would increase magnitude sensitivity with little effect on delay sensitivity. Future research should explore the role of a hypothetical delay and real reward on impulsive choice. While exposure to the experiential delay condition first resulted in improved sensitivity to delay and magnitude ratios, individuals still showed a systematic shift in making more LL choices when they completed the hypothetical delay task second. Individuals were more likely to say that they would wait to get the larger reward when they did not have to wait. This suggests that the hypothetical nature of the task was still affecting choices even after the participants calibrated their choices in response to experience with the delays.

In the current experiments, delay sensitivity and magnitude sensitivity were highly correlated. High delay sensitivity and low magnitude sensitivity are typically viewed as characteristics of impulsive choice [31,32]. The high delay sensitivity and high magnitude sensitivity observed on these experiential impulsive choice tasks suggests that those individuals are unwilling to wait for the reward, yet also want the larger reward. This finding is important to consider in relation to food choices made outside of the laboratory. Although people may decide between a small amount now and a larger amount later, there are ample situations where people can have large amounts of palatable, energy-dense food with a short delay (e.g., fast food restaurants). The combination of greater delay and magnitude sensitivity could be problematic as this pattern would promote choices of “larger-sooner” rewards.

While *k* is commonly used as a measure of delay sensitivity, *k*-values combine sensitivity to delay and magnitude [37]. Our analysis method parses delay sensitivity and magnitude sensitivity. Parsing delay sensitivity and magnitude sensitivity is important to determine the processes that might be contributing to impulsive behavior. Experiment 2 demonstrated that similar *k*-values can be obtained under very different patterns of behavior. Therefore, future research should parse delay sensitivity and magnitude sensitivity and assess their relationship to *k*-values to allow for the patterns of behavior to be better described. Human studies have made several attempts to model monetary discounting using dual-process models [38,39]. Modeling and deriving multiple parameters has several challenges including the inability to fit a model to certain individuals. Therefore, a multilevel logistic regression of choice data, as conducted in these experiments, can address the challenges of theoretical models while estimating delay sensitivity and magnitude sensitivity [32].

When comparing the current tasks to the Kirby questionnaire, the results indicated that performance on the novel impulsive choice tasks was not correlated with the *k*-values obtained from the Kirby questionnaire, consistent with previous reports [23,40]. The lack of a correlation between the novel impulsive choice tasks and the Kirby questionnaire suggests that the tasks are picking up on different processes. Hypothetical choice tasks may be more likely to reflect intentions, whereas real choice tasks should reflect actual preferences. The marshmallow test, as an example of a real choice task, is predictive of real-world outcomes most likely because it is an actual choice. However, it should be noted that the hypothetical (delay discounting) tasks, nevertheless, predict real-life behavior and may measure an impulsive endophenotype that is predictive of maladaptive tendencies. Yet, it remains important to determine how experiential tasks differ from hypothetical tasks for a variety of reasons. Experiential tasks might have superior predictive power for some aspects of real-life behavior. For example, short time-scales (e.g., minutes and seconds) might be misjudged in their effects on motivation. For example, people may fail to appreciate how aversive a 30 s wait can be, consistent with Experiment 2 results. In contrast, decisions at long time-scales (e.g., weeks, months, years as in the Kirby questionnaire) might be delayed enough to seem abstract and even hypothetical [41]. Thus, hypothetical tasks might better approximate real-world decisions that occur at longer time-scales. If experiential tasks involve different underlying decision-making mechanisms, then it is important to isolate those differences and further explore the processes underlying impulsive choice. Finally, experiential tasks in humans might be a better analog to tasks used to study impulsive choice in nonhuman animals, which could improve translational applications.

It has been proposed that experiential discounting tasks may reflect boredom proneness or sensation seeking [24]. For example, people may choose the smaller-sooner option because they are bored and want to complete the task faster. The current experiments indicated that performance on the impulsive choice tasks for food was not significantly correlated with boredom proneness and sensation seeking. This is in contrast to previous reports indicating that performance on experiential discounting tasks is correlated with boredom proneness [24], which could contribute to differences in performance across tasks. The novel impulsive choice tasks in this study were developed to address the criticisms of previous experiential tasks. It is possible that the addition of an ITI addressed the relationship with boredom proneness because adding an ITI ensures there is no large advantage to picking the SS. Participants would complete the session in about the same time regardless of which options they choose. However, further research should investigate these relationships with larger sample sizes to determine if the addition of an ITI ensures the experiential discounting tasks are measuring a new construct separate from boredom proneness. It is also important to note that sensation seeking was moderately correlated with delay sensitivity and magnitude sensitivity. A post hoc power calculation indicates that 60 and 54 participants, respectively, would be needed to detect an effect. Therefore, sensation seeking may be related to performance on the experiential discounting tasks, and future research should continue to examine this relationship with larger sample size.

The real-delay task may also be affected by time perception abilities. People are generally poor at perceiving time [42] and may focus on the reward amounts instead of the delays. Consistent with this idea, a recent study showed that individuals primarily paid attention to the amount of money, not the delay to reward, on a hypothetical impulsive choice task [43]. Because participants experienced the delays to reward in the current tasks, this may draw attention to the delays. This may explain why experiential discounting and hypothetical discounting tasks are not well correlated [23,40]. Indeed, when participants did not have to wait for the delay when they received the hypothetical task first in Experiment 2, they were not sensitive to delay. This supports research demonstrating that people are poor at perceiving time [42] and suggests that people may not be able to accurately report how the delay would affect their behavior if they do not have to experience the delay.

Altogether, we have developed an impulsive choice task for food that closely resembles the tasks used in rodents, thus improving translation between animal models and humans. The continuity of impulsive choice across species is important because it permits the application of behavioral and neurobiological research using nonhuman animal models to inform human research. In the current novel impulsive choice tasks, participants received certain rewards, consumed the rewards during the session, waited the delay, and experienced the inter-trial interval addressing several of the previous concerns of experiential discounting tasks [24]. Understanding impulsive choice for rewards with short delays provides a closer translation to animal models and may have implications for choices in real life. For example, Appelhans et al. [44] imposed a 25 s delay to unhealthy snack options, while the healthy snacks were delivered immediately, in three vending machines on the campus of a medical center. This simple intervention significantly increased healthy snack purchases. This demonstrates that the experience of real delays may impact actual food choices.

The novel impulsive choice tasks with real delays developed in the current experiments could be used in neuroscience and/or clinical studies. The real-delay, hypothetical-reward impulsive choice task could be used in imaging studies to understand how the brain processes the delays to rewards. In addition, experiential tasks may better relate to real life choices that happen on time scales in the seconds to minutes range compared to those used in the typical hypothetical impulsive choice tasks. Experiential discounting tasks, especially for real rewards, may predict impulsive food choices in real time better than hypothetical tasks. Indeed, obesity and impulsive choice are most strongly related on impulsive choice tasks for food [19]. Therefore, utilizing a real food impulsive choice task to uncover the relationship between obesity, food choice, and impulsive choice may be the most applicable. Future research should determine how impulsive choices on the current novel tasks relates to food choice, substance abuse, and other maladaptive behaviors that might be better characterized by an experiential task.

## 5. Conclusions

The current set of experiments developed a novel experiential impulsive choice task for food that more closely resembled tasks used in rodent models and addressed some of the criticisms of current experiential impulsive choice tasks. The results indicate that experiencing the delays in a task dramatically impacts impulsive choice. Performance on the novel task was similar if the participants waited the delay regardless of whether the participants experienced the reward. However, participants were less sensitive to delay and magnitude if they did not experience the delay, suggesting that they struggled to estimate how the delays would affect their choices. In addition, performance on the experiential impulsive choice tasks was not associated with self-reported measures of impulsive choice, boredom proneness, or sensation seeking. Altogether, the novel experiential impulsive choice tasks developed in these experiments measure a different construct than common survey measures, which may better predict real choices where delays are actually experienced.

## Figures and Tables

**Table 1:** Participant characteristics.

	Experiment 1	Experiment 2
Participants	50	50
Sex (M|F)	24|26	18|32
Age (min–max)	23.3 (18–34)	20.5 (18–33)

**Table 2:** Full model output for Experiment 1 from a logistic regression with the fixed effects of task (RD/HR = 1; RD/RR = −1), delay ratio (log-transformed), and magnitude ratio (log-transformed). RD/RR = real-delay, real-reward. RD/HR = real-delay, hypothetical-reward. SE = standard error.

	Estimate (*b*)	SE	*z*	*p*
Delay Ratio	3.82	0.55	6.97	<0.001
Magnitude Ratio	−4.37	0.43	−10.23	<0.001
Delay Ratio × Task	0.38	0.24	1.62	0.11
Magnitude Ratio × Task	−0.06	0.12	−0.46	0.65

**Table 3:** Full model output for Experiment 2 from a logistic regression with the fixed effects of task (RD/HR = 1; HD/HR = −1), order (RD/HR first = 1; HD/HR first = −1), delay ratio (log-transformed), and magnitude ratio (log-transformed). RD/HR = real-delay, hypothetical-reward. HD/HR = hypothetical-delay, hypothetical-reward.

	Estimate (*b*)	SE	*z*	*p*
Delay Ratio	2.39	0.47	5.08	<0.001
Magnitude Ratio	−3.49	0.47	−7.41	<0.001
Delay Ratio × Task	2.06	0.23	8.83	<0.001
Delay Ratio × Order	0.65	0.45	1.45	0.15
Magnitude Ratio × Task	−0.48	0.11	−4.23	<0.001
Magnitude Ratio × Order	−0.94	0.46	−2.06	0.04
Delay Ratio × Task × Order	−0.49	0.12	−4.19	<0.001
Magnitude Ratio × Task × Order	0.57	0.11	4.98	<0.001

**Table 4:** Delay sensitivity, magnitude sensitivity, and *k* derived from the logistic regression (logistic *k*) for each task depending on the task order. The task listed under Order is the first task experienced. RD/HR = real-delay, hypothetical-reward. HD/HR = hypothetical-delay, hypothetical-reward.

Order	Task	Delay Sensitivity	Magnitude Sensitivity	Logistic *k*
RD/HR	RD/HR	4.61	−4.35	1.06
	HD/HR	3.53	−4.52	0.78
HD/HR	RD/HR	4.28	−3.61	1.19
	HD/HR	1.24	−1.49	0.83

**Table 5:** Correlation matrix for Experiment 1. Logistic *k* and Kirby *k* were log-transformed.

	1	2	3	4
**1 Delay Sensitivity**	1.00			
**2 Magnitude Sensitivity**	−0.65 **	1.00		
**3 Logistic *k***	0.27	0.41 *	1.00	
**4 Kirby *k***	−0.14	0.17	0.14	1.00

* *p* < 0.05, ** *p* < 0.001.

**Table 6:** Correlation matrix for Experiment 2. Logistic *k* and Kirby *k* were log-transformed.

	1	2	3	4	5	6
**1 Delay Sensitivity**	1.00					
**2 Magnitude Sensitivity**	−0.92 **	1.00				
**3 Logistic *k***	0.02	0.23	1.00			
**4 Kirby *k***	0.16	−0.26	−0.22	1.00		
**5 Boredom Proneness**	−0.13	0.23	0.27	−0.07	1.00	
**6 Sensation Seeking**	0.35	−0.37	−0.002	0.26	0.23	1.00

** *p* < 0.001.

## Appendix A

Table A1 displays the individual items for the experiential impulsive choice tasks developed in this manuscript.

**Table A1:** Individual items for the experiential impulsive choice tasks. The smaller-sooner (SS) and larger-later (LL) delays were in seconds. Items 1–13 were experienced in the first half of the session (Block 1) and items 14–25 were experienced in the second half (Block 2). Within each block, the items were delivered in a random order.

Item	SS Amount	LL Amount	SS Delay	LL Delay	Delay Ratio	Mag Ratio
1	1	5	5	25	0.2	0.2
2	2	4	5	25	0.2	0.5
3	4	5	5	25	0.2	0.8
4	2	5	10	25	0.4	0.4
5	3	5	10	25	0.4	0.6
6	1	5	15	30	0.5	0.2
7	2	4	15	30	0.5	0.5
8	4	5	15	30	0.5	0.8
9	2	5	15	25	0.6	0.4
10	3	5	15	25	0.6	0.6
11	1	5	20	25	0.8	0.2
12	2	4	20	25	0.8	0.5
13	4	5	20	25	0.8	0.8
14	2	5	5	25	0.2	0.4
15	3	5	5	25	0.2	0.6
16	1	5	10	25	0.4	0.2
17	2	4	10	25	0.4	0.5
18	4	5	10	25	0.4	0.8
19	2	5	15	30	0.5	0.4
20	3	5	15	30	0.5	0.6
21	1	5	15	25	0.6	0.2
22	2	4	15	25	0.6	0.5
23	4	5	15	25	0.6	0.8
24	2	5	20	25	0.8	0.4
25	3	5	20	25	0.8	0.6

## References

[1] Ainslie G.W. (1974). Impulse control in pigeons. J. Exp. Anal. Behav..

[2] MacKillop J., Amlung M.T., Few L.R., Ray L.A., Sweet L.H., Munafò M. (2011). Delayed reward discounting and addictive behavior: A meta-analysis. Psychopharmacology.

[3] Alessi S.M., Petry N.M. (2003). Pathological gambling severity is associated with impulsivity in a delay discounting procedure. Behav. Process..

[4] Amlung M., Petker T., Jackson J., Balodis I., MacKillop J. (2016). Steep discounting of delayed monetary and food rewards in obesity: A meta-analysis. Psychol. Med..

[5] Scheres A., Sumiya M., Thoeny A.L. (2010). Studying the relation between temporal reward discounting tasks used in populations with ADHD: A factor analysis. Int. J. Methods Psychiatr. Res..

[6] Wilson V.B., Mitchell S.H., Musser E.D., Schmitt C.F., Nigg J.T. (2011). Delay discounting of reward in ADHD: Application in young children. J. Child Psychol. Psychiatry.

[7] Bickel W.K., Athamneh L.N., Basso J.C., Mellis A.M., DeHart W.B., Craft W.H., Pope D. (2019). Excessive discounting of delayed reinforcers as a trans-disease process. Curr. Opin. Psychol..

[8] Bickel W.K., Jarmolowicz D.P., Mueller E.T., Koffarnus M.N., Gatchalian K.M. (2012). Excessive discounting of delayed reinforcers as a trans-disease process contributing to addiction and other disease-related vulnerabilities: Emerging evidence. Pharmacol. Ther..

[9] Schlam T.R., Wilson N.L., Shoda Y., Mischel W., Ayduk O. (2013). Preschoolers’ delay of gratification predicts their body mass 30 years later. J. Pediatrics.

[10] Rachlin H., Raineri A., Cross D. (1991). Subjective probability and delay. J. Exp. Anal. Behav..

[11] Mischel W., Ebbesen E.B. (1970). Attention in delay of gratification. J. Personal. Soc. Psychol..

[12] Odum A.L. (2011). Delay discounting: I’m ak, you’re ak. J. Exp. Anal. Behav..

[13] Mazur J.E., Commons M.L., Mazur J.E., Nevin J.A. (1987). An adjusting procedure for studying delayed reinforcement, in Quantitative analyses of behavior. The Effect of Delay and of Intervening Events on Reinforcer Value.

[14] Bickel W.K., Mueller E.T. (2009). Towards the study of trans-disease processes: A novel approach with special reference to the study of co-morbidity. J. Dual Diagn..

[15] Madden G.J., Begotka A.M., Raiff B.R., Kastern L.L. (2003). Delay discounting of real and hypothetical rewards. Exp. Clin. Psychopharmacol..

[16] Robertson S.H., Rasmussen E.B. (2018). Comparison of potentially real versus hypothetical food outcomes in delay and probability discounting tasks. Behav. Process..

[17] Lawyer S.R., Schoepflin F., Green R., Jenks C. (2011). Discounting of hypothetical and potentially real outcomes in nicotine-dependent and nondependent samples. Exp. Clin. Psychopharmacol..

[18] Green R.M., Lawyer S.R. (2014). Steeper delay and probability discounting of potentially real versus hypothetical cigarettes (but not money) among smokers. Behav. Process..

[19] Barlow P., Reeves A., McKee M., Galea G., Stuckler D. (2016). Unhealthy diets, obesity and time discounting: A systematic literature review and network analysis. Obesity Rev..

[20] Rasmussen E.B., Lawyer S.R., Reilly W. (2010). Percent body fat is related to delay and probability discounting for food in humans. Behav. Process..

[21] Schiff S., Amodio P., Testa G., Nardi M., Montagnese S., Caregaro L., di Pellegrino G., Sellitto M. (2015). Impulsivity toward food reward is related to BMI: Evidence from intertemporal choice in obese and normal-weight individuals. Brain Cogn..

[22] Shiels K., Hawk L.W., Reynolds B., Mazzullo R.J., Rhodes J.D., Pelham W.E., Waxmonsky J.G., Gangloff B.P. (2009). Effects of methylphenidate on discounting of delayed rewards in Attention Deficit/Hyperactivity Disorder. Exp. Clin. Psychopharmacol..

[23] Reynolds B., Schiffbauer R. (2004). Measuring state changes in human delay discounting: An experiential discounting task. Behav. Process..

[24] Smits R.R., Stein J.S., Johnson P.S., Odum A.L., Madden G.J. (2013). Test–retest reliability and construct validity of the Experiential Discounting Task. Exp. Clin. Psychopharmacol..

[25] Kirby K.N., Petry N.M., Bickel W.K. (1999). Heroin addicts have higher discount rates for delayed rewards than non-drug-using controls. J. Exp. Psychol. Gen..

[26] Holt S.H.A., Brand Miller J.C., Petocz P., Farmakalidis E. (1995). A satiety index of common foods. Eur. J. Clin. Nutr..

[27] Wang X.T., Dvorak R.D. (2010). Sweet future: Fluctuating blood glucose levels affect future discounting. Psychol. Sci..

[28] Kirby K.N. (2009). One-year temporal stability of delay-discount rates. Psychon. Bull. Rev..

[29] Farmer R., Sundberg N.D. (1986). Boredom proneness: The development and correlates of a new scale. J. Personal. Assess..

[30] Zuckerman M., Eysenck S., Eysenck H. (1978). Sensation seeking in England and America: Corss-cultural, age, and sex comparisons. J. Consult. Clin. Psychol..

[31] Wileyto E.P., Audrain-McGovern J., Epstein L.H., Lerman C. (2004). Using logistic regression to estimate delay-discounting functions. Behav. Res. Methods Instrum. Comput..

[32] Young M.E. (2018). Discounting: A practical guide to multilevel analysis of choice data. J. Exp. Anal. Behav..

[33] Burnham K.P., Anderson D.R. (2002). Model Selection and Multimodel Inference: A Practical Information-Theoretic Approach.

[34] Holm S. (1979). A simple sequentially rejective multiple test procedure. Scand. J. Stat..

[35] Steele C.C., Pirkle J.R., Davis I.R., Kirkpatrick K. (2019). Dietary effects on the determinants of food choice: Impulsive choice, discrimination, incentive motivation, preference, and liking in male rats. Appetite.

[36] Steele C.C., Pirkle J.R.A., Kirkpatrick K. (2017). Diet-induced impulsivity: Effects of a high-fat and a high-sugar diet on impulsive choice in rats. PLoS ONE.

[37] Young M.E. (2017). Discounting: A practical guide to multilevel analysis of indifference data. J. Exp. Anal. Behav..

[38] Peters J., Miedl S.F., Büchel C. (2012). Formal Comparison of Dual-Parameter Temporal Discounting Models in Controls and Pathological Gamblers. PLoS ONE.

[39] Price M., Higgs S., Maw J., Lee M. (2016). A dual-process approach to exploring the role of delay discounting in obesity. Physiol. Behav..

[40] Reynolds B., Richards J.B., de Wit H. (2006). Acute-alcohol effects on the Experiential Discounting Task (EDT) and a question-based measure of delay discounting. Pharmacol. Biochem. Behav..

[41] Yi R., Stuppy-Sullivan A., Pickover A., Landes R.D. (2017). Impact of construal level manipulations on delay discounting. PLoS ONE.

[42] Eagleman D.M. (2008). Human time perception and its illusions. Curr. Opin. Neurobiol..

[43] Amasino D.R., Sullivan N.J., Kranton R.E., Huettel S. (2019). Amount and time exert independent influences on intertemporal choice. Nat. Hum. Behav..

[44] Appelhans B.M., French S.A., Olinger T., Bogucki M., Janssen I., Avery-Mamer E.F., Powell L.M. (2018). Leveraging delay discounting for health: Can time delays influence food choice?. Appetite.

